# Supplementary figures and images for: Characterisation of the role and regulation of Ultrabithorax in sculpting fine-scale leg morphology
Source: Front Cell Dev Biol. 2023 Feb 13;11:1119221. doi: 10.3389/fcell.2023.1119221 (PMC9968978; doi:10.3389/fcell.2023.1119221)

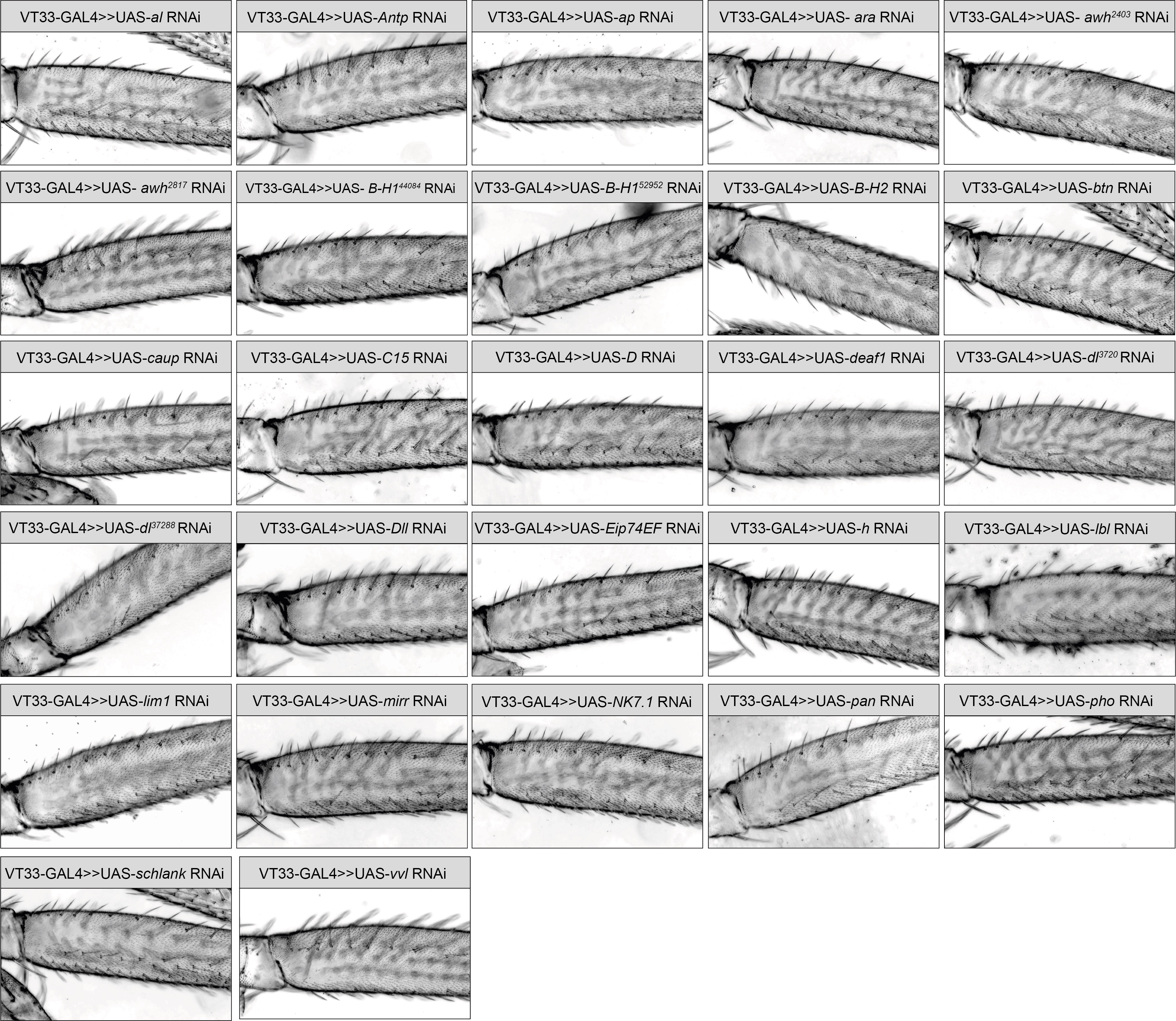

Supplement: Supplementary file 1 [file Image3.JPEG]

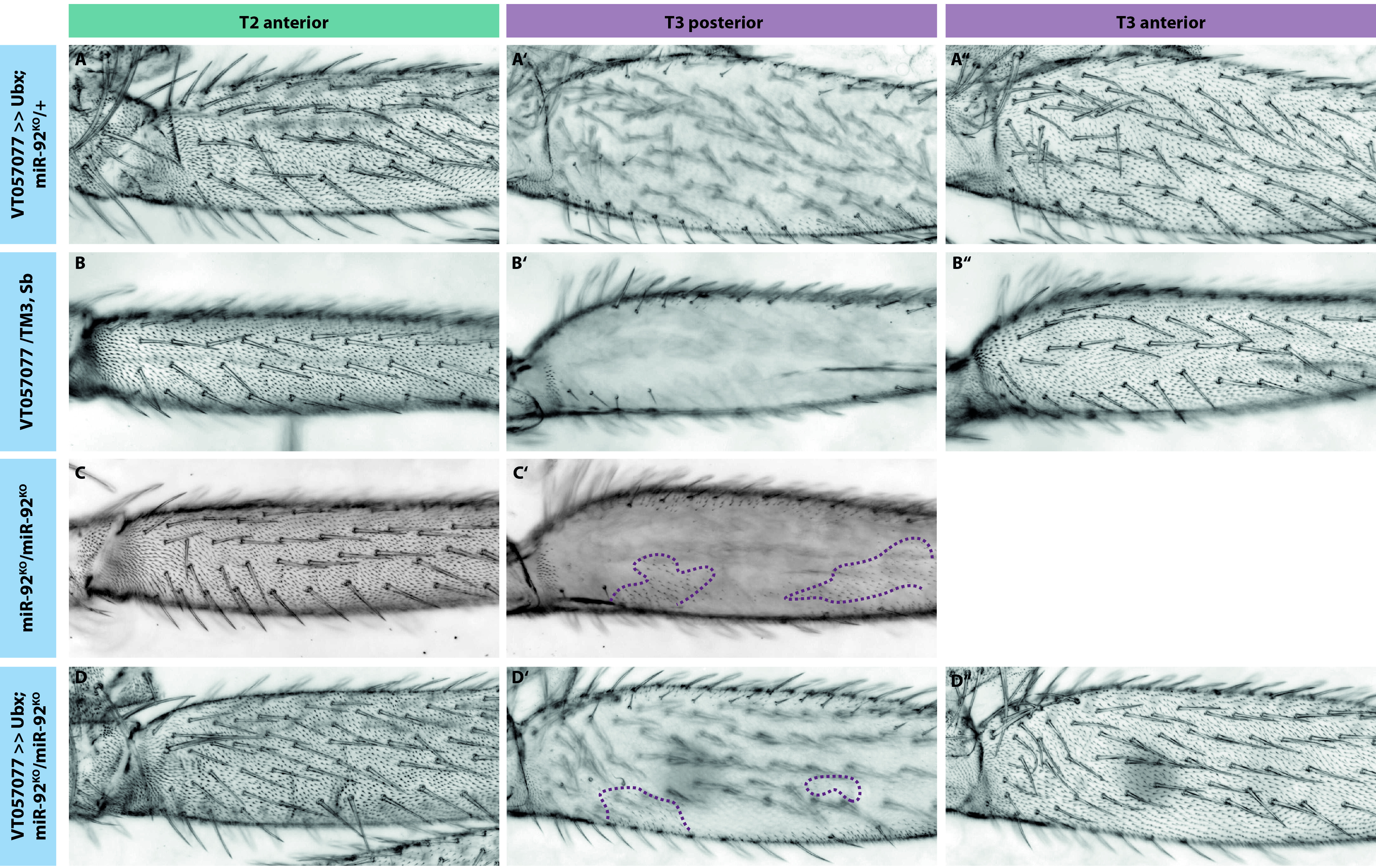

Supplement: Supplementary file 3 [file Image1.JPEG]

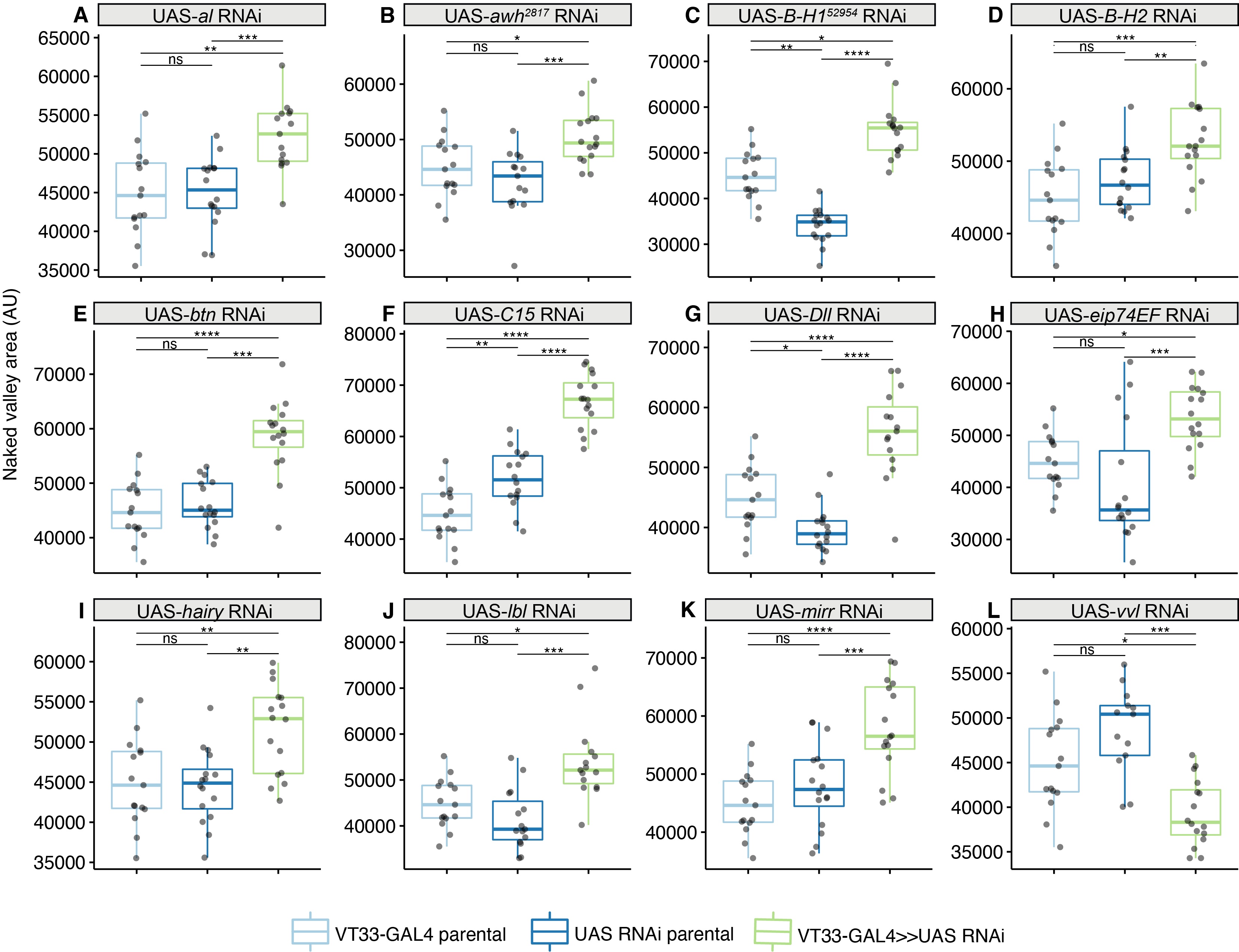

Supplement: Supplementary file 4 [file Image4.JPEG]

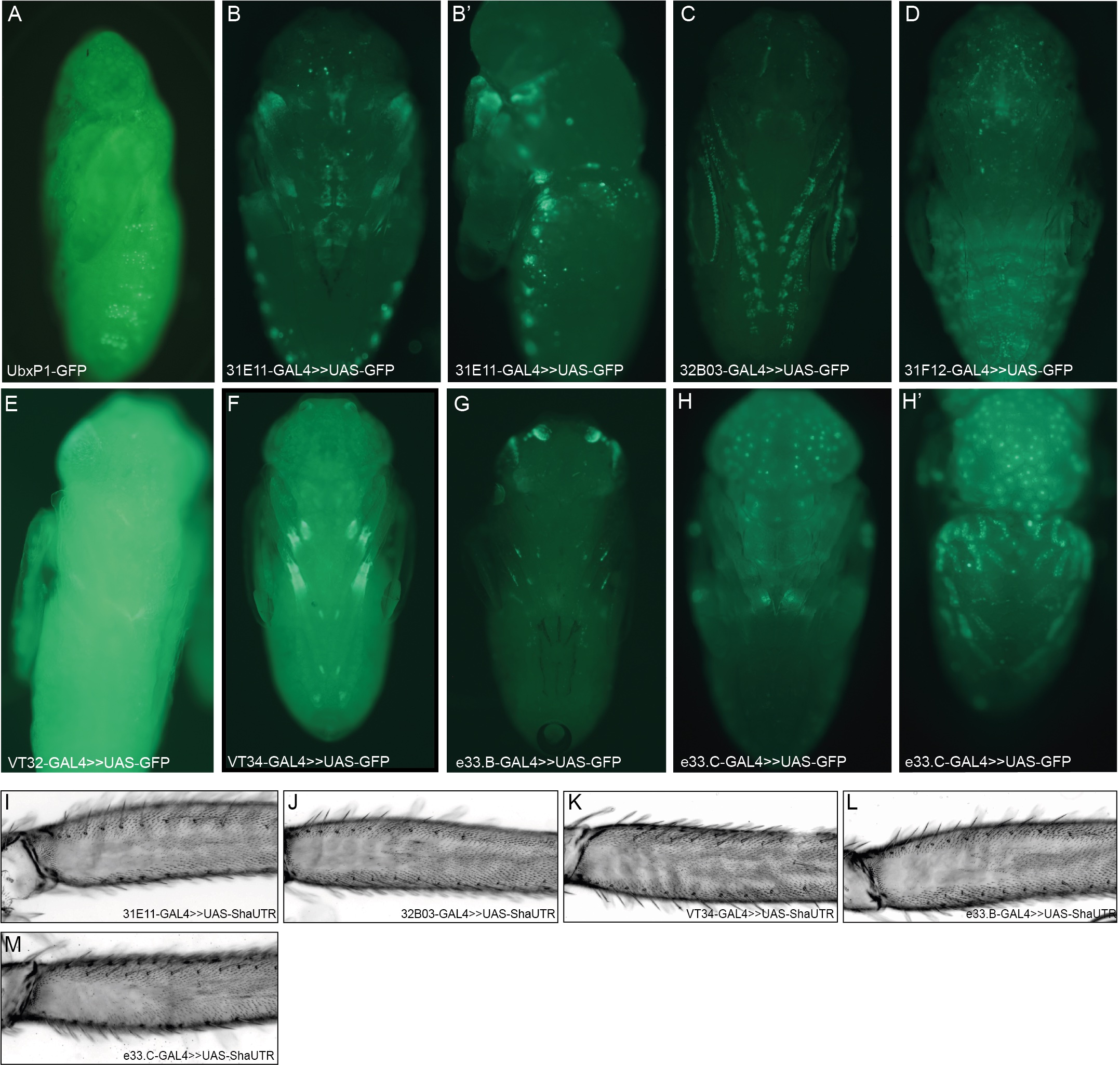

Supplement: Supplementary file 5 [file Image2.JPEG]

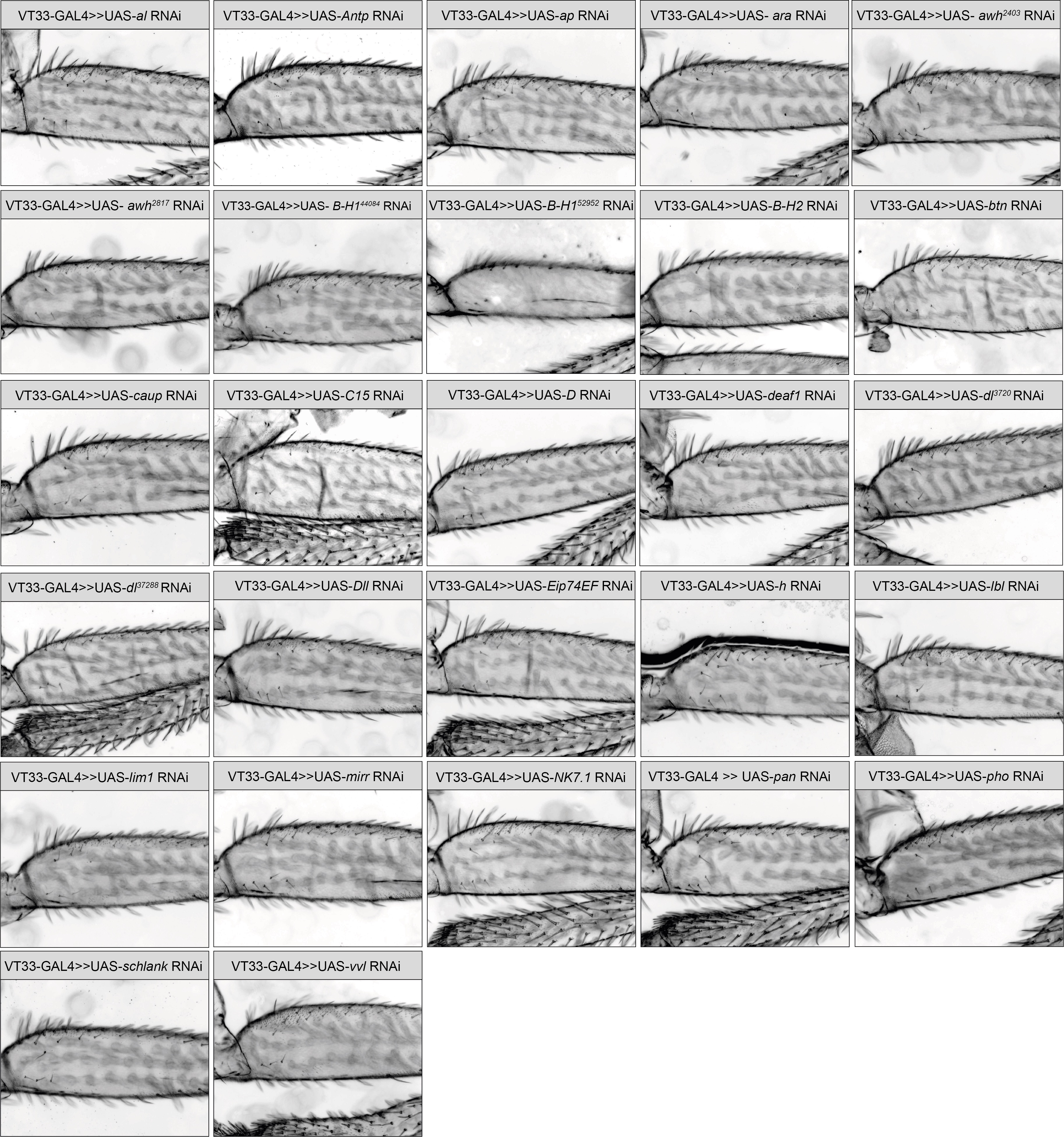

Supplement: Supplementary file 6 [file Image5.JPEG]
